# NEC is likely a NETs dependent process and markers of NETosis are predictive of NEC in mice and humans

**DOI:** 10.1038/s41598-018-31087-0

**Published:** 2018-08-22

**Authors:** Deirdre Vincent, Michaela Klinke, Georg Eschenburg, Magdalena Trochimiuk, Birgit Appl, Bastian Tiemann, Robert Bergholz, Konrad Reinshagen, Michael Boettcher

**Affiliations:** 1 Department of Pediatric Surgery, University Medical Center Hamburg-Eppendorf, Martinistrasse 52, 20246 Hamburg, Germany; 2 Department of Experimental Animal Research, University Medical Center Hamburg-Eppendorf, Martinistrasse 52, 20246 Hamburg, Germany

## Abstract

Necrotizing enterocolitis (NEC) is one of the most devastating diseases affecting premature and mature infants. It is hypothesized that NEC is the result of neutrophils’ active role in hyperinflammation after bacterial gut colonization, through their nuclear DNA release and formation of neutrophil extracellular traps (NETs) to combat pathogens. The aim of this study was to evaluate the importance of NETs in NEC pathogenesis, as well as to identify and validate markers of NETosis to predict NEC. NEC was induced in mice by gavage feeding of Neocate and lipopolysaccharide, followed by ten minutes of hypoxia (5% O2) q12h for five days, starting on day four postpartum (p.p.). The interrelation of NEC and neutrophils, including NETs, was assessed macroscopically (i.e. NEC score, SYTOX Orange), microscopically (i.e. Chiu score, citrullinated histone H3, neutrophil elastase), and in blood samples (i.e. cell-free DNA (cfDNA), DNase). In order to determine the exact role of NETs in NEC pathogenesis, a protein arginine deiminase (PAD) inhibition model was established (preventing NETs formation in mice) by injecting BB-Cl-amidine once daily, starting on day one p.p. Additionally, human intestinal samples of diagnostically verified NEC were analyzed. In total, 76 mice were analyzed in the experiment. Serum cfDNA correlated positively with NEC manifestation, as measured by macroscopic NEC score (r = 0.53, p = 0.001), and microscopic evaluation with Chiu score (r = 0.56, p < 0.001). Markers of neutrophil activation and NETosis were significantly increased in animals with NEC and in human samples as compared to controls. Further, prevention of NETosis by protein arginine deiminase (PAD) inhibition in mice significantly reduced mortality, tissue damage, and inflammation in mice induced with NEC. Our results suggest that the hyperinflammation observed in NEC is a NETs-dependent process, as NEC severity was significantly reduced in mice incapable of forming NETs (PAD inhibition) and markers for NEC and NETs correlated positively during the time course of NEC induction. Further, serum surrogate markers of NETosis (such as cfDNA and DNase) appear to predict NEC in neonatal mice. As findings of the mouse NEC model correlate positively with human NEC samples immunohistochemically, the hyperinflammation reaction observed in mice could potentially be applied to human NEC pathogenesis.

## Introduction

Necrotizing enterocolitis (NEC) is considered one of the most devastating diseases affecting premature and term-born infants, as its manifestation often leads to necrosis of the small intestine, which results in mortality rates of up to 30% in very low birth weight (VLBW) infants^1^. Moreover, it affects approximately 12% of infants with a birth weight of less than 1500 g^2^.

Despite its high prevalence and years of research on the topic, the pathogenesis remains unclear. However, current findings not only suggest that NEC is a multifactorial disease process, but also, that it is almost always preceded by bacterial exposure of the immature intestine after birth^3^.

Upon exposure, the premature infant develops a local hyperinflammation response, which ultimately damages the neonatal intestine and results in NEC development^4^. Thereafter, the intestine of term infants’ has been shown to adapt to the hyperinflammation via a downregulation of their immune system; whereas, in preterm infants, no downregulation occurs^3^.

One of the main mechanism supporting this theory, on a cellular level, is the activity of the intestinal epithelial cell toll-like receptor 4 (TLR4), which exhibits a higher expression in the preterm intestine than in the intestine of term-born infants. This upregulation of TLR4 leads to increased mucosal injury, as: (1) apoptosis of enterocytes becomes accelerated and (2) the rate of healing is reduced through impaired intestinal restoration and proliferation^3^. Additionally, numerous studies, including clinical trials, have strengthened the case for TLR4′s role in the pathogenesis of NEC. One such example is the protective effect of breastfeeding on NEC development, in that it inhibits the signaling cascade that leads to TLR4 activation^3,5^. This increased signaling cascade activity, caused by a higher TLR4 expression, results in the production of chemokines and inflammatory cytokines that are necessary for the recruitment of neutrophils to the location of inflammation^6,7^. Based on previous research findings, we hypothesized that recruitment of neutrophils must also play a crucial role in NEC development. Neutrophils, as activated by the neonatal innate immune system, defend against infectious agents as first line responders and eliminate pathogens by phagocytosis and/or degranulation^8^. However, excessive neutrophil activity is widely known to cause tissue damage, especially during acute inflammatory responses^9^. This type of damage is more notable in human neonatal intestinal epithelial cells in comparison to adult intestinal epithelial cells, as demonstrated by an inflated production of cytokines in response to pathogens, inflammatory mediators, and commensal bacteria in neonates. The consequence is an excessive neutrophil migration to the area of inflammation^4^. Upon activation, neutrophils combat pathogens using three main mechanisms: (1) phagocytosis, (2) degranulation, and (3) formation of neutrophil extracellular traps (NETs)^10^.

The relatively novel mechanism of NET formation describes the process by which neutrophils release web-like DNA structures studded with histones and antimicrobial proteins. To date, two mechanisms of NET formation are known. The first and main pathway constitutes apoptosis of the neutrophil in question and is also known as NETosis. The second mechanism involves NET formation without apoptosis, thus enabling neutrophils to continue functioning^11^. NETosis via cell death occurs two to four hours after stimulation, whereas NET formation occurs more rapidly, approximately five to 60 minutes after stimulation^11,12^. When formed, NETs have several functions. Two of significance to this study are: (1) they fight infection through immobilizing microbes, such as bacteria, fungi, viruses, and protozoa in their DNA mesh; and (2) they eliminate microorganisms via lethal concentrations of antimicrobial agents contained within the NETs^13–15^.

As the role of NETs in NEC development has yet to be investigated, the aim of this study was to determine whether NETs play a crucial role in NEC pathogenesis. Therefore, both (1) a NEC mouse model and (2) human NEC intestinal samples were evaluated. Additionally, in order to determine the extent of NETs’ contribution to NEC development, a mouse model using protein arginine deiminase (PAD) inhibition was employed to prevent NETs from being formed.

## Methods

### Study design

The study was approved by the Hamburg State Administration for animal research (63/16). A total of 76 mice were included in the experimental model and were held within the animal facility, according to environmental parameters established and dictated by the German guide for the care and use of laboratory animals (Tierschutzgesetz).

Additionally, histological intestinal samples of nine human neonates that underwent surgery due to NEC at the Department of Pediatric Surgery of the University Medical Center Hamburg-Eppendorf from 2016 to 2017 were included in the study. Only cases with confirmed classical NEC, without prior patent ductus arteriosus closure, or complex health conditions, were included. Examinations were in accordance with the guidelines of the medical research ethics committee of Hamburg (Ethik-Kommission der Ärztekammer Hamburg, PV4991) and with the 1964 Helsinki declaration and its later amendments. Written informed consent was obtained from the parents or legal guardians of the nine neonates.

### Animal Procedures

#### NEC Induction Paradigm

NEC was induced using a well-established protocol^16^: Pregnant C57BL/6 J mice were singly housed within the animal care facility with food and water ad lib. Mother animals delivered naturally and pups were kept with their mother throughout the entire experimental procedure. On day four postpartum (p.p.), the NEC induction paradigm commenced. Subjects were gavage fed a solution of 0.1 ml Neocate (Nutricia) and lipopolysaccharide (LPS) from E. coli LPS-EB (InvivoGen), using a 1:50 concentration of LPS to Neocate twice daily followed by 10 minutes of hypoxia at 5% oxygen. The total induction paradigm was maintained for six days and the animals were euthanized on day 10 p.p., at the latest.

### NEC and NETs Timeframe

**Figure 1:**
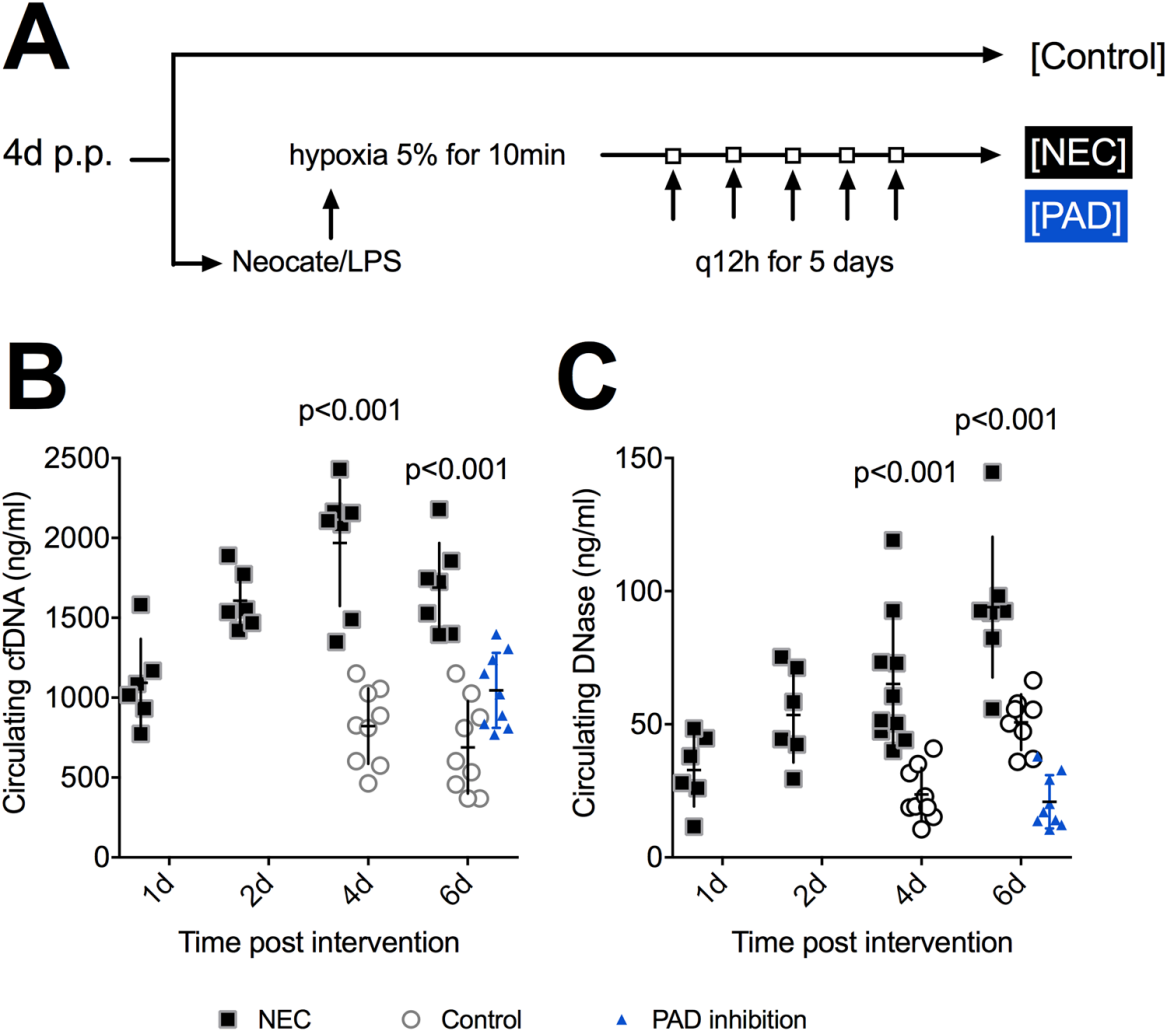
Experimental design and biomarkers of necrotizing enterocolitis (NEC). (**A**) Mice within the NEC group were subjected to Neocate/lipopolysaccaride gavage feeding followed by hypoxia at 5% for 10 min twice daily for five days. The induction was started on day four postpartum (p.p.). All animals were euthanized on day 10 p.p. at the latest. (**B**) Cell-free (cfDNA) is a surrogate marker of NETosis. It significantly increased with time and compared to controls. Protein arginine deiminase (PAD) inhibition (blue) resulted in significantly lower levels of cfDNA in comparison to untreated animals. (**C**) DNase showed a near linear significant increase with time. Animals with NEC had significantly higher levels than controls and PAD inhibited mice (blue). Data shown as Mean ± SD. Statistics: Mann-Whitney test and ANOVA.

The induction protocol is shown in Fig. 1A. To assess the development of NEC and NETs, animals were euthanized via decapitation after sedation with isoflurane gas (Forene 100%, AbbVie) at the following endpoints: 1d, 2d, 4d, and 6d after induction commencement. During the entire length of the experiment, mother animals received tramadol (Tramal 1 mg/ml, Grünenthal) in their drinking water for pain management of the pups. Tramadol ingested by mother mice is transferred to offspring pups through drinking of breastmilk at 12–14% of its original concentration, which has been shown to ensure appropriate pain management for test subjects^17^. To control for other factors that may influence the pups, a control group was also included.

### PAD-Inhibition

Protein Arginine Deiminase 4 (PAD4) is a histone-modifying enzyme and its inhibition, by either genetic knockout or Cl-amidine treatment, has been shown to prevent NET formation^18^. In this study, PAD inhibition was achieved by daily subcutaneous injections of 10 mg/kg bodyweight Cl-amidine (BB-Cl-amidine, Cayman Chemical, preparation according to manufacturer’s protocol), starting on day one p.p. for the entirety of the procedure^19^. Keeping in accordance with the NEC induction paradigm, PAD inhibited mice underwent NEC induction on day four p.p., as described in the previous section, while controlling for pain through mother-pup-transfer of tramadol via breast milk until euthanasia. All animals were euthanized on day 10 p.p. Figure 1A demonstrates the time frame outlining the PAD inhibition paradigm.

#### Sample Collection and Storage

Blood: Upon euthanasia, blood samples were collected through decapitation in test tubes containing ethylenediaminetetraacetic acid and processed immediately. The samples were then centrifuged at 2000 relative centrifugal force for 10 minutes at room temperature, followed by plasma collection and separation from the buffy coat. All samples were preserved at a temperature of −80 °C until further analysis.

Intestinal Tissue: After blood collection, animals were dissected using a midline incision. Bowel preparation and removal was conducted with aid of a light microscope to better evaluate the intestine for macroscopic markers of NEC. At this time, the morphologic analysis was performed and captured using a 4 K/12-megapixel camera. The relevant segments of the small intestine were then evenly distributed into test tubes containing: (1) phosphate buffered saline (PBS) and (2) Bouin solution. Tissue stored in PBS was used directly to visualize NETs.

#### Blood Analysis

Blood plasma was used to assess levels of cell free DNA (cfDNA) and DNase in individual subjects, where cfDNA is considered a surrogate marker for NET formation and DNase represents the degradation of cfDNA. Plasma was collected and analyzed for cfDNA, as well as DNase, based on previously described and validated methods^20^.

#### Morphologic Analysis of the Intestine

Scoring of macroscopic NEC manifestation occurred during dissection and was performed by two observers blinded to the subject’s test group. As none of the animals showed necrosis or perforation, animals were simply divided into (0) No NEC or (1) NEC, according to whether they did not or did demonstrate pneumatosis intestinalis or infarction, respectively.

#### Tissue Preparation and Evaluation

Extracellular DNA: Intestinal samples were placed in PBS and analyzed for NETs under a fluorescence microscope using SYTOX Orange (50 µM, Life Technologies)^21^. Pictures were taken using an Olympus SC 50 camera and digitized with cellSens Standard (Olympus). Scoring was conducted by an observer blinded to the study as followed:

- None (0) – no signs of NETs
- Mild (1) – small amount of NETs
- Moderate (2) – medium amount of NETs
- Severe (3) – large amount of NETs

Histological Analysis: Upon tissue fixation in Bouin solution, intestinal samples were dehydrated overnight and embedded in paraffin. Prepared intestinal tissue was then cut into 3 µm thick sections and applied to slides for further analysis. All samples were analyzed by a pathologist blinded to the study and captured using an Olympus SC 50 camera. Digitization was completed via the cellSens Standard program (Olympus).

Hematoxylin and Eosin (H&E): H&E staining occurred via machine and using a standardized staining procedure, while semi-quantitative assessment of the intestinal damage was conducted under light microscopy, utilizing the previously validated Chiu score^22^.

Immunohistochemical Staining (NE, MPO, H3cit, TRL4): Histological samples were further immunohistochemically stained for neutrophil elastase (NE), myeloperoxidase (MPO), citrullinated histone H3 (H3cit), and TLR4: Neutrophil activation and recruitment was evaluated with NE (ABCAM, ab68672) and MPO (DIANOVA, DLN-012930) staining procedures; while NETs production was assessed with H3cit (ABCAM, ab5103), a surrogate marker for NETs; and inflammation via TLR4 (ABCAM, ab22048) staining. Subsequently, the stained samples were incubated according to manufacturer’s instructions. In accordance with each antibody examined, an appropriate isotype control antibody was used as a negative control. Digitized MPO, NE, H3cit, and TLR4 immunohistochemically stained slides were then evaluated in a semi-quantitative fashion:

- None (0) – no signs of tissue staining
- Mild (1) – small amount of tissue staining
- Moderate (2) – medium amount of tissue staining
- Severe (3) – large amount of tissue staining

TUNEL Assay: A TUNEL assay was performed to detect DNA fragmentation in cell nuclei (a marker for apoptosis in intestinal tissue) using an *In Situ* Cell Death Detection Kit (Roche, Mannheim, Germany). The amount of apoptosis was assessed in a semi-quantitative fashion by an observer blinded to the study groups:

- None (0) – no signs of tissue staining
- Mild (1) – small amount of tissue staining
- Moderate (2) – medium amount of tissue staining
- Severe (3) – large amount of tissue staining

ELISA (C5a): In order to assess recruitment of inflammatory cells, such as neutrophils, a complement component 5a (C5a) ELISA using plasma was employed. The chemoattractant C5a is hypothesized to be a main factor involved in the inflammation cascade causing NEC and, as such, was measured in this study using a standard C5a KIT (Abcam, Cambridge, UK), according to manufacturer’s instructions^23^. The results are expressed as pg/ml.

Human Sample Collection: Blood and intestinal samples were collected at the time of surgery upon removal of necrotic tissue. In order to be included in the study, NEC diagnosis had to be confirmed surgically by the attending surgeon. The intestinal samples were stained according to the previous description of mice sample preparation (HE, NE, MPO, H3Cit, TLR4) and analyzed by a pathologist blinded to the study.

Statistics: All data was analyzed using SPSS Statistics 24 (IBM, NY, USA) and GraphPad Prism 7 (GraphPad, CA, USA). A pre-power study calculation was performed using G*Power 3.1. The power was deducted from a previous study examining NEC in mice^24^. Differences between groups were calculated using the ANOVA test (with planned contrasts) and results are presented as mean ± standard deviation (SD). For ordinal data, differences were calculated with the Mann-Whitney test, while association between factors was statistically evaluated using Spearman’s Rho. The level of significance for all tests was set at <0.05.

## Results

**Figure 2:**
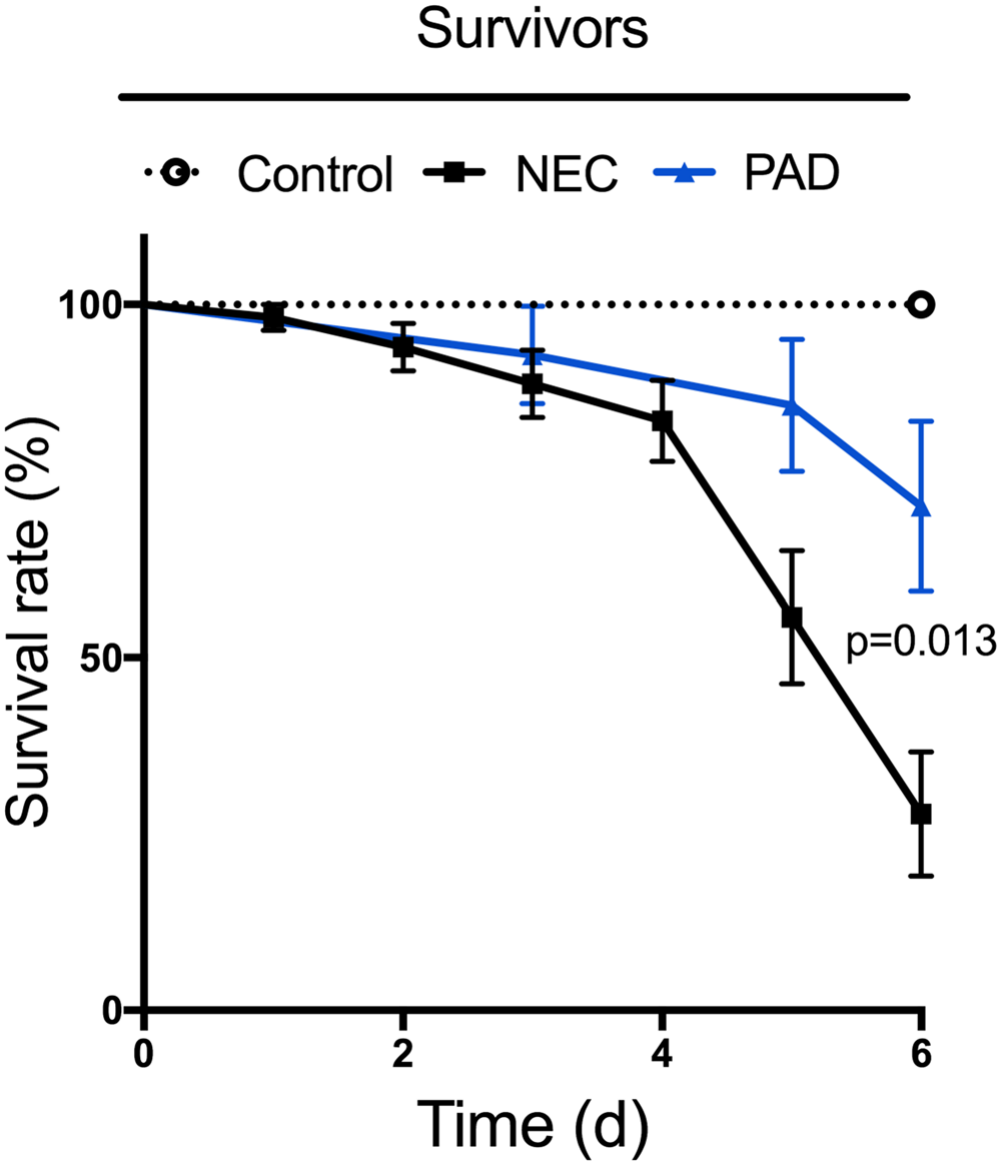
Survival rates of animals after NEC induction. Less than 30% of animals in the intervention group survived until their predetermined date of euthanasia. Animals with PAD inhibition (blue) were subjected to the same NEC induction protocol but had a significantly reduced mortality rate compared to animals in the NEC group (p = 0.013). Statistics: Wilcoxon test.

Survival rates are shown in Fig. 2, with a total of 51 out of 76 animals surviving the entirety of the procedure. Specifically, all animals in the control group survived until day six (10/10), while only 50% of animals in the NEC group reached this endpoint (7/14). Animals that underwent PAD inhibition demonstrated an increased survival rate, with 10 out of 14 animals reaching this endpoint. Moreover, the mortality rate at the predetermined endpoint of day 10 p.p. was significantly reduced after PAD inhibition (p = 0.0129) and resembled control mice mortality rates. Regarding NEC manifestation, the majority of animals within the NEC induction group showed clinical signs of NEC, such as abdominal distension. In line with these clinical signs, NEC manifestation increased with time: 1d (0/6), 2d (1/8), 4d (4/10), 6d (5/7), based on macroscopic inspection. In comparison to the NEC group, animals that underwent PAD inhibition, had a significantly lower and especially less severe NEC manifestation score at the final endpoint (4/10 versus 5/7, p = 0.003).

Surrogate markers of NET production, namely cfDNA and DNase, are shown in Fig. 1B,C, where cfDNA increased notably with time (p = 0.007) and levels were significantly higher within the NEC groups than in controls (Fig. 1B). Additionally, ROC analysis (comparing mice after NEC induction to controls at day four and six of NEC induction start) showed 100% sensitivity and specificity for cfDNA levels above 1250 ng/ml (p = 0.001). DNase concentration also significantly increased with time, most likely as a consequence of NET production and tissue damage (p = 0.002), and animals with NEC exhibited significantly higher DNase levels than control animals (Fig. 1C). In contrast, animals with PAD inhibition had significantly lower cfDNA and DNase levels compared to NEC mice (Fig. 1B,C). Compared to age-matched controls however, cfDNA levels in PAD inhibited mice were significantly higher (p = 0.02), while their DNase concentrations were markedly lower (p < 0.001). This trend can be explained by the hypoxia treatment that PAD inhibited mice received, as hypoxic states have been shown to increase cfDNA levels. Thus we can assume that NETosis prevention in PAD inhibited mice was successful and that the measured increase in cfDNA levels in mice with PAD inhibition is most likely a consequence of tissue damage caused by the hypoxia treatment and not induced by NETosis^25^. Ultimately, a moderate positive correlation was observed between cfDNA and DNase levels (R = 0.57, p < 0.001), reflecting an upregulation of DNase in mice with high cfDNA levels.

**Figure 3:**
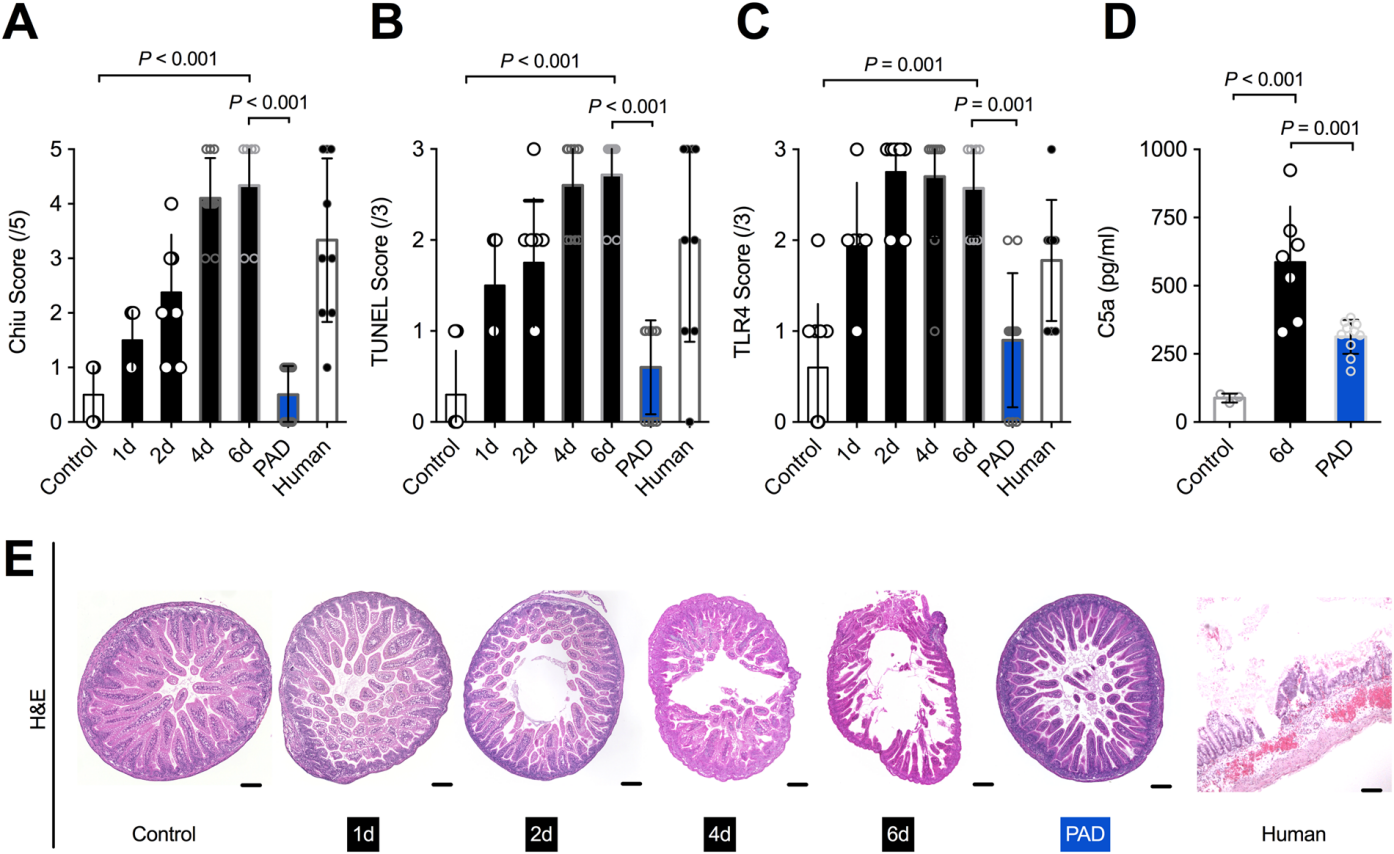
PAD inhibition reduces tissue damage and apoptosis. (**A,E**) Using the Chiu score for assessment, there was a significant increase in intestinal tissue damage with time that was prevented in mice undergoing PAD inhibition (blue). These findings are illustrated by staining the samples with hematoxylin & eosin. (**B**) Apoptosis was assessed using a TUNEL assay. Apoptosis was vastly increased after NEC induction, while animals with PAD inhibition (blue) showed significantly lower TUNEL scores than animals of the NEC group. (**C,F**) Toll like receptor 4 (TRL4) expression increased with time in animals with NEC in comparison to control animals. PAD inhibition (blue) prevented this effect. (**E**) Complement activation was assessed via a complement component 5a (C5a) assay, which showed a linear significant elevation in concentration after NEC induction. This increase in C5a was prevented by PAD inhibition (blue). Total number of subjects: controls n = 10, 1d NEC n = 6, 2d NEC n = 8, 4d NEC n = 10, 6d NEC n = 7, PAD inhibition n = 10, humans n = 9. Statistics: Mann-Whitney test and ANOVA.

Microscopic evaluation of NEC manifestation demonstrated a significant increase in tissue damage within the NEC group in comparison to controls (p < 0.001). More specifically, animals undergoing NEC induction had significantly higher Chiu scores than controls, as shown in Fig. 3A,E. Furthermore, apoptosis rates increased drastically with time amongst NEC group animals (p < 0.001) and TUNEL scores were notably higher in the NEC group than in controls, as seen in Fig. 3B. In comparison to the NEC group, animals with PAD inhibition appeared to be protected from tissue damage and displayed significantly less apoptosis (Fig. 3A,B).

General inflammation and complement activation, as measured by TLR4 expression and C5a activation, respectively, were more pronounced with increased length of the NEC induction paradigm. In fact, TLR4 expression was very high in all animals undergoing NEC induction (Fig. 3C) and showed a steady increase with time (p > 0.05). In contrast, mice with PAD inhibition showed significantly less TLR4 expression (Fig. 3C). Moreover, animals within the NEC group exhibited a severe complement activation, which was reduced by PAD inhibition (Fig. 3D).

**Figure 4:**
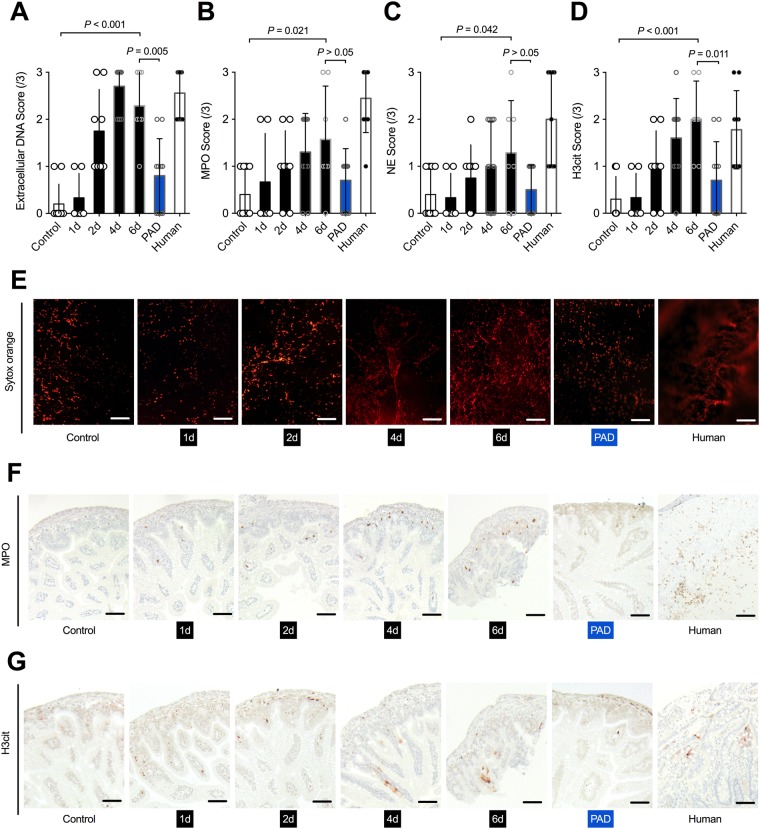
NEC induces neutrophil activation and possibly NET formation. (**A,E**) Quantification of extracellular DNA, including neutrophil extracellular traps (NETs), was visualized by SYTOX Orange staining. NEC animals demonstrated a relevant increase in NETs, which was significantly reduced in the absence of PAD (blue). Human subjects with NEC had similar NET scores. (**B,C,F**) Neutrophil elastase (NE) and myeloperoxidase (MPO), both markers of neutrophil activation, increased with time in NEC animals in comparison to controls, and were reduced in animals with PAD inhibition (blue). (**D,G**) Quantification of the NET marker citrullinated histone H3 (H3cit) showed an elevation in animals, as well as humans with NEC. The surrogate marker was significantly reduced in the PAD inhibition group (blue), with H3cit levels in animals with PAD inhibition being close to controls (p = 0.075). Total number of subjects: controls n = 10, 1d NEC n = 6, 2d NEC n = 8, 4d NEC n = 10, 6d NEC n = 7, PAD inhibition n = 10, humans n = 9. Statistics: Mann-Whitney test and ANOVA.

As predicted, NEC induction resulted in severe neutrophil activation and NETs production. In fact, extracellular DNA significantly increased with time amongst NEC group animals (p = 0.002), and animals with NEC revealed considerably higher levels of extracellular DNA in comparison to controls (Fig. 4A,E). PAD inhibited animals had significantly lower levels than controls (Fig. 4A). Further, neutrophil activation, as assessed by MPO and NE, significantly increased with time (MPO p = 0.034; NE p = 0.018) and levels were markedly higher than in controls (Fig. 4B,C). Again, animals with PAD inhibition displayed a non-significant reduction in neutrophil activation (Fig. 4B,C). Additionally, NETs production, as measured by H3cit, significantly increased with time (p < 0.001) while control animals displayed considerably lower levels, as seen in Fig. 4D. Likewise, PAD inhibition appeared to have been successful, as these animals had significantly lower H3cit scores than their NEC counterparts (Fig. 4D).

### Human Subjects

All subjects were prematurely born (27.56 + /−2.24 SSW), underweight (1038.89 + /−317.99 g), and underwent surgery between day nine to 10 p.p. (9.57 + /−8.46d). As shown in Fig. 3, human NEC intestinal samples experienced similar levels of tissue damage (Chiu score), apoptosis (TUNEL) and inflammation (TLR4), as compared to mice with NEC. Moreover, neutrophil activation, measured by MPO and NE, as well as neutrophils undergoing NETosis, were comparable between human and mice NEC samples, as seen in Fig. 4.

## Discussion

The exact pathophysiology of NEC remains unclear, but immaturity of the intestinal barrier coupled with an exaggerated inflammatory response, initiated by the immature intestinal epithelium in response to injury, is the leading hypothesis^14,26,27^. At sites of inflammation, neutrophils eliminate pathogens via: (1) phagocytosis and (2) through the production of web-like DNA structures that are coated with histones and proteolytic enzymes, which are known as NETs^28^. These NETs may be the key to understanding NEC pathogenesis. In fact, the current study demonstrated that NEC manifestation was mostly prevented in PAD inhibited mice, which were incapable of producing NETs, when subjected to the same NEC induction protocol as NEC mice. Even more, all aspects commonly associated with NEC manifestation, such as tissue damage, apoptosis, and inflammation were significantly reduced in animals with PAD inhibition. Therefore, as PAD4 is a necessary enzyme required for the process of NETosis, and thus, animals with PAD inhibition were unable to produce NETs^29^, it is very likely that NETs contribute crucially to NEC pathogenesis.

The results of the current study support the hyperinflammation hypothesis that explains NEC development. Statistical analysis of our research findings revealed that after NEC induction, animals showed a robust neutrophil activation and an increase in NETs-producing neutrophils, which is in line with previous studies^30,31^. Moreover, animals with NEC showed a strong activation of (1) the complement system and (2) an increased expression of TLR4, both a result of activated neutrophils. On one hand, neutrophils not only induce NETosis, but also activate the complement signaling cascade, which in turn results in C5a production^32^. Additionally, NETs induce and/or enhance complement system activation via i.e. activation of Factor XII, which subsequently leads to neutrophil recruitment and NETosis^33^. On the other hand, increased expression of TLR4 in NEC has been reported previously in both mice and humans^3,34^. This can be explained by TLR4s’ role in facilitating the recognition of invading pathogens by responding to bacterial DNA and LPS^35^. Even more, activation of TLR4 has been shown to result in a release of pro-inflammatory cytokines (i.e. interleukin 6, tumor necrosis factor alpha) via MyD88-dependent pathways and to initiate platelet aggregation^36^. Thus, considering these two cascades, initiated by activated neutrophils, one can state that NETs, as produced by neutrophils during NEC, induce complement activation and further inflammation through increased TLR4 expression with subsequent activation of additional neutrophils. Hence, the inflammatory cascade observed in NEC, results in a vicious cycle in which neutrophils induce hyperinflammation via production of NETs and these NETs further activate neutrophils. Thus, we postulate that NETs are essential for NEC progression. Further, as our human NEC samples showed similar levels of neutrophil activation and NET production, it is conceivable that the results of the animal model are transferable to human NEC patients.

Adding to the role of NETs in NEC, our findings suggest that NET markers, like DNase and cfDNA, may serve as means to diagnose NEC. Recent studies support our findings as these also showed the presence of cfDNA and neutrophilic proteins in the intestine of neonates with NEC^30^. However, the pool of cfDNA is likely made up of DNA of various origins, including: (1) the formation of NETs by neutrophils, and (2) passive release of mitochondrial and nuclear DNA of injured and dying cells^26,37–40^. Thus, not all extracellular DNA identified by staining procedures in this study is composed of NETs only. However, cfDNA concentrations were significantly increased in animals undergoing NEC induction in comparison to controls in this study. This is in line with current research demonstrating that human neonates suffering from NEC experience high levels of cfDNA (above 1000 ng/ml)^41^. Even though the study did not result in pronounced cfDNA levels, all NEC mice displayed cfDNA concentrations above 1250 ng/ml, which distinguished all NEC animal subjects from control mice. Nevertheless, before cfDNA can be adopted as a diagnostic marker for NEC, further studies examining other diseases of the intestine are needed.

Regarding results of the PAD inhibition mouse model, we believe that differences between PAD inhibited mice and normal physiological mice could have been even more pronounced, if PAD4 inhibition would have been effectuated using a genetic knockout model. Therefore, using an inhibition model and not a knockout model could be considered a potential limitation of this study. However, with this in mind, the H3cit expression in the PAD inhibition group of the current study was almost equivalent to baseline levels, as seen in the control group. Thus, we are convinced that the PAD4 function was almost entirely blocked within this group. Strengthening our PAD inhibition model, are previous studies that showed excellent results of Cl-amidine in order to achieve PAD4 inhibition^18^. In fact, C57BL/6 J mice might not be the optimal animal subject for NEC research, as neutrophil levels in humans are more than two times higher than in mice^37,38^. It is thus possible that NETs might even play a more substantial role in NEC pathogenesis than observed in this current mouse NEC model.

In conclusion, our study supports the theory that NETs play an essential part in NEC pathogenesis. Overall, NEC mortality and, in particular morbidity were vastly reduced after inhibition of NETosis through PAD inhibition, with levels mimicking those of healthy controls not subjected to the NEC induction protocol. As such, NETs appear to be vital to the hyperinflammation cascade accompanying NEC development and we believe that inhibition of NETosis might limit the consequences of NEC. Furthermore, cfDNA appears to be an excellent marker for diagnosis of NEC. However, further studies are needed to validate our findings.
